# Baseline factors associated with death in a COVID-19 hospital cohort, Sao Paulo, 2020

**DOI:** 10.11606/s1518-8787.2021055003684

**Published:** 2021-11-08

**Authors:** Thais Claudia Roma de Oliveira Konstantyner, Camila Bertini Martins, Carla Gianna Luppi, Suely Miyuki Yashiro, Nívia Aparecida Pissaia Sanches, Frederico Molina Cohrs, Paulo Abrão Ferreira, Eduardo Alexandrino Medeiros

**Affiliations:** I Universidade Federal de São Paulo Escola Paulista de Medicina Departamento de Medicina Preventiva São PauloSP Brasil Universidade Federal de São Paulo. Escola Paulista de Medicina. Departamento de Medicina Preventiva. São Paulo, SP, Brasil; II Universidade Federal de São Paulo Escola Paulista de Medicina Departamento de Medicina São PauloSP Brasil Universidade Federal de São Paulo. Escola Paulista de Medicina. Departamento de Medicina. São Paulo, SP, Brasil

**Keywords:** COVID-19, mortality, Hospital Mortality, Risk Factors, Socioeconomic Factors, Cohort Studies

## Abstract

This study aimed to verify socio-demographic and baseline clinical factors associated with death in a hospital cohort of patients with COVID-19. A retrospective cohort study was conducted between February and December 2020 in a university hospital in the city of São Paulo, using Hospital Epidemiology Center data. RT-PCR-positive patients were selected to compose the sample (n = 1,034). At the end of the study, 362 (32%) patients died. In this cohort, age equal to or greater than sixty years (HR = 1.49) and liver disease (HR = 1.81) were independent risk factors for death from COVID-19 associated with higher in-hospital mortality.

## INTRODUCTION

The coronavirus disease 2019 (COVID-19) was first identified in December 2019, in Wuhan, the capital of China’s Hubei province, and has since spread globally, resulting in the ongoing pandemic. Until December 31, 2020, approximately 83 million cases and 1.8 million deaths due to COVID-19 were reported worldwide.

In Brazil, 7,619,2000 cases and 193,875 deaths were reported in the official systems of the Brazilian Ministry of Health in the same period. The State of São Paulo remains the one with the highest numbers: 1,462,297 cases and 46,717 deaths; approximately 33% of these occurred in the city of São Paulo.

The spectrum of this disease ranges from mild to life-threatening. Some cases might progress promptly to acute respiratory distress syndrome and/or multiple organ function failure. The presence of some specific social conditions, individuals’ characteristics, comorbidities, and clinical aspects of the disease have been identified as predictors of death, making research on these themes crucial to prevent the event.

Thus, our study aimed to verify socio-demographic and baseline clinical factors associated with death in a hospital cohort of patients with COVID-19.

## METHODS

A retrospective cohort study was conducted with hospitalized patients with COVID-19 between February and December 2020, in Hospital São Paulo, a university hospital in the city of São Paulo.

We used the data collected systematically by the Hospital Epidemiology Center, using a standardized form for data collection on the severe acute respiratory syndrome, according to clinical-epidemiological criteria established by the Brazilian Ministry of Health (available from: https://opendatasus.saude.gov.br/dataset/bd-srag-2020). The study population was composed of suspected COVID-19 hospitalized patients (n = 2,540). Patients with confirmation of COVID-19 infection by the polymerase chain reaction test (RT-PCR) were selected to compose the sample of the present analysis (n = 1,095). Patients with unknown clinical evolution (cure or death) were excluded from the sample (n = 65). Thus, the final sample was composed of 1,034 hospitalized patients with COVID-19.

A descriptive analysis of the cohort was conducted. The Kaplan-Meier product-limit estimator was used to estimate the cumulative probability curves for the date of first onset of symptoms and the date of clinical evolution concerning the independent variables. The log-rank test was used for the comparison of the curves (data not showed).

The Cox regression model for survival analysis was used to investigate factors associated with death. In the model, death by COVID-19 was considered an event and the cure as censure. The hazard ratio (HR) was estimated for each independent variable. The Wald test was used to test the hypothesis of HR = 1. The null hypothesis was rejected when the p-value was ≤ 0.05. The variables that maintained statistical significance and those that statistically adjusted the parameters of the other variables remained in the final Cox model. Proportionality of risk over time was determined using Schoenfeld’s residual analysis, which employs a chi-square statistic with one degree of freedom based on the proportion of observed and expected survival. All analyses were conducted using R program.

This study received approval from the Human Research Ethics Committee (CAAE no. 37940720.5.0000.5505).

## RESULTS

Of the 1,034 patients analyzed, most were male (59%), 58% had 9 or fewer years of education level and a half of the cohort were 60 years of age or older. Concerning the comorbidities, 90% had at least one and cardiovascular disease was the most prevalent (45%). The most frequent symptoms observed were oxygen saturation < 95% (80%), dyspnea (77%), cough (73%), respiratory discomfort (70%), and fever (66%). The length of stay mean was 17 days (IQR: 12–26 days). At the end of the study, 362 (32%) patients died.

In the adjusted model, age equal to or greater than sixty years and the presence of liver disease were independent risk factors for death from COVID-19, while the presence of fever at the time of admission was negatively associated with the event.

The Table shows the complete distribution of patients according to variables of interest and the univariate and multivariate analyses of factors associated with death.

**Table:** Distribution of patients according to demographic and clinical characteristicsa, bivariate and multivariate analysesb of factors associated with death in a hospital cohort of patients with COVID-19. Hospital São Paulo, February to December, 2020.

Characteristic	n (%)	HR	p	HR adj^c^	p
Sex					
Female	426 (41%)	-	-	-	-
Male	608 (59%)	0.98	0.828	-	-
Age					
< 60 years	513 (50%)	-	-	-	-
≥ 60 years	521 (50%)	2.01	< 0.001	**1.49**	**0.006**
Race					
White	555 (56%)	-	-	-	-
Black	104 (10%)	0.82	0.300	-	-
Yellow	23 (2%)	1.01	> 0.900	-	-
Brown	314 (32%)	1.05	0.700	-	-
Education level					
≤ 9 years	367 (58%)	-	-	-	-
> 9 years	262 (42%)	0.70	0.007	0.76	0.058
Symptoms					
Fever	678 (66%)	0.75	0.008	**0.74**	**0.035**
Cough	756 (73%)	0.82	0.073	-	-
Sore troath	131 (13%)	0.83	0.319	-	-
Dispynea	788 (77%)	1.41	0.018	1.25	0.370
Respiratory discomfort	715 (70%)	1.31	0.031	1.09	0.718
Oxigen saturation < 95%	808 (80%)	1.4	0.029	1.11	0.609
Diarrhea	234 (23%)	0.86	0.227	-	-
Vomiting	152 (15%)	0.93	0.625	-	-
Other symptom	549 (55%)	0.92	0.432	-	-
Comorbidity					
Cardiovascular disease	418 (45%)	1.54	< 0.001	1.27	0.102
Hematological disease	45 (5%)	1.38	0.188	-	-
Liver disease	59 (6%)	1.83	0.001	1.81	0.006
Asthma	35 (4%)	0.99	0.969	-	-
Diabetes mellitus	358 (39%)	0.91	0.377	-	-
Neurological disease	76 (8%)	1.41	0.043	1.07	0.775
Lung disease	92 (10%)	1.24	0.173	-	-
Immunodeficiency/suppression	215 (23%)	0.99	0.904	-	-
Kidney disease	254 (28%)	1.13	0.277	-	-

^a^ Number of patients analyzed = 1,034.

^b^ Number of patients analyzed = 556.

^c^ Hazard ratio adjusted (multivariate analyses).

## DISCUSSION

Our study presents three main results. First, 90% of the patients had at least one comorbidity at the time of admission and only liver disease was associated with death in this sample. Second, age greater than or equal to sixty years was independently associated with death. Finally, having a fever at the time of hospital admission was a protective factor against death by COVID-19.

The characterization of our sample was remarkably similar to that found at the beginning of the epidemic and in the largest study published on hospital admissions by COVID-19 in Brazil^1^: most patients were men, older adults, with a high prevalence of comorbidity and frequent presence of fever, cough, dyspnea, and low oxygen saturation. Cardiovascular disease was the most prevalent comorbidity in our sample, following the pattern found elsewhere in the world^2^.

Older adults and people of any age who have comorbidities are known to be more severely affected and had a higher mortality rate by COVID-19. Our analysis corroborates other studies that showed a worse prognosis in older adult population^3^. In our sample, liver disease was the only comorbidity independently associated with death. Studies have already shown that people with pre-existing liver disease who were diagnosed with COVID-19 are at higher risk than people without the disease^4^. Finally, in our analysis, the presence of fever was negatively associated with death by COVID-19. The fact that the person has a fever would be an indication of a better immune competence to fight the virus^5^.

Our study has limitations and strengths that should be considered. It is crucial to note that this was a hospital-based study, with data from a university hospital, with specific care characteristics. This fact could impact the representativeness of our findings, although the characterization of our sample was remarkably similar to other studies as mentioned above. Moreover, the Brazilian Ministry of Health’s standardized form contained some missing data for reported symptoms and comorbidities, which made it impossible to analyze characteristics previously associated with death, such as obesity.

On the other hand, our study had an expressive number of patients analyzed and its period covers the entire year of 2020. This can bring important information about the clinical characteristics and factors associated with death in the period of the pandemic when the new variants of the virus were not yet circulating effectively in the city of São Paulo and vaccination had not yet been implemented.

We concluded that age greater than or equal to sixty years and liver disease were associated with higher in-hospital mortality in a cohort of patients with COVID-19 admitted to a university hospital in the city of São Paulo. Knowing the populations with the highest risk of death by COVID-19 is crucial for the implementation of prevention therapeutic strategies, and the organization of the hospital service.
